# *Sanguisorba officinalis* L synergistically enhanced 5-fluorouracil cytotoxicity in colorectal cancer cells by promoting a reactive oxygen species-mediated, mitochondria-caspase-dependent apoptotic pathway

**DOI:** 10.1038/srep34245

**Published:** 2016-09-27

**Authors:** Meng-ping Liu, Min Liao, Cong Dai, Jie-feng Chen, Chun-juan Yang, Ming Liu, Zuan-guang Chen, Mei-cun Yao

**Affiliations:** 1 School of Pharmaceutical Sciences, Sun Yat-sen University, Guangzhou 510006, P. R. China; 2 College of Pharmacy, Harbin Medical University, Harbin 150081, P. R. China

## Abstract

*Sanguisorba officinalis* L. radix is a widely used herb called DiYu (DY) in China and has an extensive range of bioactivities, including anti-cancer, anti-inflammatory, and anti-oxidative activities. However, there is little evidence to support its anti-cancer effects against colorectal cancer (CRC). The first-line chemotherapeutic agent 5-fluorouracil (5-FU) is used to treat CRC, but its efficiency is hampered by acquired drug resistance. This study found that a water extract of DY exerted anti-proliferative effects against two CRC cell lines (HCT-116 and RKO), and it sensitized CRC cells to 5-FU therapy by activating a reactive oxygen species (ROS)-mediated, mitochondria-caspase-dependent apoptotic pathway. Co-treatment of DY and 5-FU significantly elevated ROS levels, up-regulated Bax/Bcl-2 ratio and triggered mitochondrial dysfunction, followed by a release of cytochrome c and up-regulation of proteins such as cleaved-caspase-9/3 and cleaved-PARP. Additionally, the induction of autophagy may be involved in mediating synergism of DY in HCT-116 cells. Gallic acid (GA), catechinic acid (CA) and ellagic acid (EA) were identified as the potential chief constituents responsible for the synergistic effects of DY. In conclusion, co-treatment of DY, specifically GA, CA and EA, with 5-FU may be a potential alternative therapeutic strategy for CRC by enhancing an intrinsic apoptotic pathway.

Colorectal cancer (CRC) remains a serious threat to human health and contributes to a third of cancer-related deaths worldwide^1^. Despite significant advances in therapeutic strategies for CRC, the 5-year survival of patients is less than 12.5%. Acquired drug resistance is regarded as a major reason for 90% of the failures of cancer therapy^2^. The first-line chemotherapeutic agent 5-fluorouracil (5-FU) is extensively used alone or in combination with other chemotherapeutic agents on clinic for CRC therapy and promotes cell death by inhibiting RNA or DNA synthesis^3^. Unfortunately, drug resistance to 5-FU has been detected in tumour therapy. An increasing number of studies have demonstrated that dysfunctional apoptosis may be involved in the drug resistance to 5-FU^4^, such as the mutation of p53 or Bcl-2 family proteins^5^,^6^. To sensitize CRC cells to the cytotoxicity of 5-FU, drugs that induce apoptosis are often included in the 5-FU combined regimens^7^.

In Asia, the root of *Sanguisorba officinalis* L. (Rosaceae), referred to as great burnet, is traditionally used as an anti-inflammatory, anti-oxidative, and haematopoietic agent. Recently, several studies have shown that DY has anti-cancer activities, such as anti-proliferative effects against breast cancer, oral cancer and prostate cancer^8^,^9^,^10^. The bioactive components of DY are believed to be tannins^11^ and saponins^12^. For example, ziyuglycoside II, a major saponin in DY, exhibited cytotoxicity towards breast cancer cells by inducing apoptosis and cell cycle arrest via a ROS-mediated JNK pathway^12^. Gallic acid and ellagic acid are the major constituents of DY exerting the anti-proliferative effects against breast cancer cells by activating apoptotic pathways^8^. In spite of its wide use on clinic DY has been rarely reported to exhibit anti-cancer effects towards CRC. According to the theory of channel tropism in traditional Chinese medicine, DY is predominantly distributed to the colon and liver. Furthermore, several tannin components of DY, such as gallic acid^13^, ellagic acid^14^ and procyanidins^15^, exhibit or synergistically enhance 5-FU cytotoxicity towards CRC cells.

Therefore, we hypothesized that DY exhibits anti-proliferative effects against CRC cells, and it may enhance the cytotoxicity of 5-FU via an apoptosis-related pathway. Heat shock protein 90 (HSP90) is a molecular chaperone that is involved in cancer survival and is a promising target for cancer therapy^16^; thus, we also explored whether the synergism of DY and 5-FU was due to a cooperative block of HSP90. The potential bioactive constituents of DY that account for its anti-cancer effects were investigated as well in the present study.

## Results

### The cytotoxicity and synergism of DY against CRC cells

**Figure 1:**
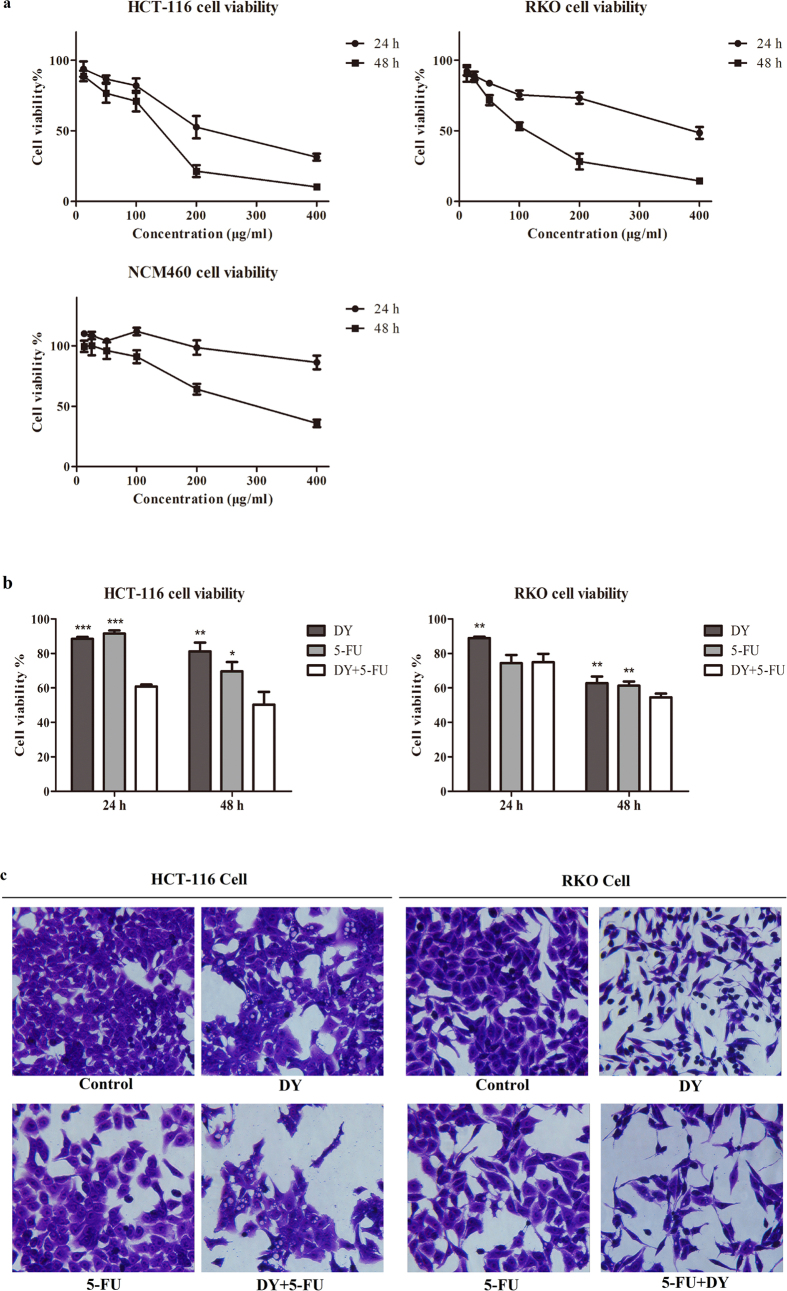
DY exhibited anti-proliferation effects against CRC cells and sensitized them to the cytotoxicity of 5-FU. (**a**) Cells were treated with DY extracts (12.5–400 μg/ml) for 24 or 48 h, CCK8 assay was then performed to evaluate the cell viability. (**b**) Cells were separately incubated with DY (100 μg/ml) or 5-FU (20 μM, HCT-116; 5 μM, RKO) and their combination for 24 or 48 h and cell viabilities were assessed by CCK8 assay. *P < 0.05, **P < 0.01, ***P < 0.001, compared with (DY + 5-FU) group. Results are expressed as mean ± SD (n = 3). (**c**) Cellular morphologies of CRC cells with 48 h treatments as described in (**b**) were observed under inverted microscope (400×).

To assess the cytotoxicity of DY in CRC cells, the cell viabilities of two CRC cell lines treated with DY extracts were determined. IC_50_ values of DY at 48 h in HCT-116 and RKO cells were 123.92 ± 46.92 μg/ml and 120.43 ± 26.59 μg/ml (IC_50_ < 200 μg/ml), respectively. While DY showed low cytotoxicity, with an IC_50_ of 255.70 ± 44.12 μg/ml, towards NCM460 cells (a normal human colorectal mucosal epithelial cell line) (Fig. 1a). Notably, the data indicate that DY had anti-proliferative activities towards two CRC cell lines.

Based on the cytotoxicity of DY towards two CRC cell lines, we then investigated the potential synergism of DY with 5-FU therapy. Cancerous and normal human colorectal cell lines were treated with DY and/or 5-FU. The data from CCK8 assays were processed by CompuSyn software as described by Chou *et al.*^17^ to calculate the combination index (CI) and the dose-reduction index (DRI). DY synergistically enhanced the cytotoxicity of 5-FU against CRC cells (CI < 1). In contrast, DY exhibited an antagonistic effect on NCM460 cells (CI > 1) (Table 1). Overall, DY may selectively improve the cytotoxicity of 5-FU in CRC cells and protect normal cells.

For further research, we combined DY and 5-FU at concentrations resulting in 30–40% inhibition to conduct the synergistic experiments. As shown in Fig. 1b, the combination of DY and 5-FU exerted stronger inhibitory effects on the viabilities of two CRC cells than a single drug (48 h).

The morphological changes of HCT-116 and RKO cells following different treatments were observed after crystal violet staining and are shown in Fig. 1c. After being exposed to DY/5-FU for 48 h, HCT-116 cells became shrank, and many cytoplasm vacuoles were observed inside. As to RKO cells, they became thinner and longer. After treatment with a combination of DY and 5-FU, lower cellular confluence and dysfunctional morphologies were observed in both cell lines.

### Synergistic anti-proliferative effects of DY and 5-FU against CRC cells were induced by apoptosis

**Figure 2:**
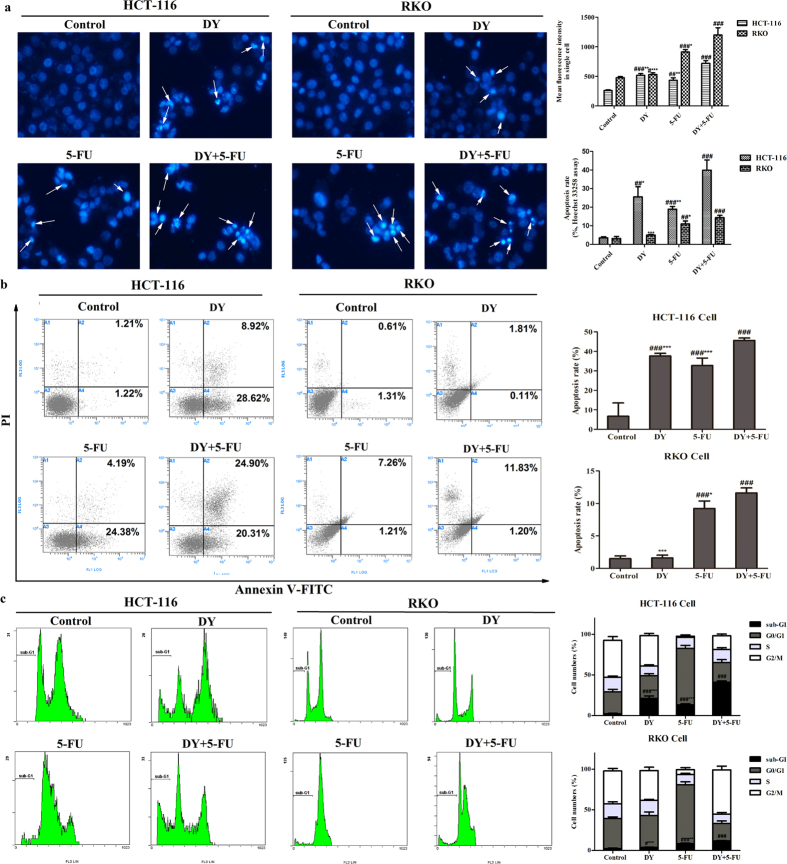
DY synergistically induced apoptosis in CRC cells when treated with 5-FU. (**a**) Cells treated with DY and/or 5-FU for 48 h were loaded with Hoechst 33258 probes, and cells with condensed or fragmentary nucleus (arrow) were observed under a fluorescence microscope (200×). The mean fluorescence intensity of Hoechst 33258 in single cell and apoptotic rates were quantified (see right histograms) as well. (**b**) Apoptotic CRC cells stained with Annexin V-FITC/PI were detected by flow cytometry system (FCMS), and apoptotic rates are showed in right bars. (**c**) Cells were stained by PI & RNase and DNA contents were then measured by FCMS. Cells arrested in the sub-G1 phase are considered to undergo late apoptosis. All data are expressed as mean ± SD (n = 3). ^**#**^P < 0.05, ^**##**^P < 0.01, ^**###**^P < 0.001, vs control group. While *P < 0.05, **P < 0.01, ***P < 0.001, vs (DY + 5-FU) group.

The chemotherapeutic drug 5-FU has been widely used on clinic for CRC therapy. Agents that enhance apoptosis are likely to suppress drug resistance to 5-FU^18^. Therefore, we determined whether the synergism between DY and 5-FU is resulted from increased apoptosis. Data of Hoechst 33258 assay shows that condensed and bright chromatin, as well as some cell debris were observed in the DY-treated HCT-116 cells (Fig. 2a), indicating that apoptosis was induced by DY in HCT-116 cells. However, DY only slightly induced apoptosis in RKO cells at the given dose. When cells were treated with a combination of 5-FU and DY, the mean fluorescence intensity in single cell remarkably increased, which were observed in both cell lines (Fig. 2a). To confirm the above results, Annexin V-FITC/PI and PI & RNase staining assays were then conducted. Following treatment with the DY and 5-FU combination, the percentages of apoptotic cells were 45.59 ± 1.27% and 11.61 ± 0.78% in HCT-116 and RKO cells, respectively, which were dramatically higher than those in the single treatment groups (Fig. 2b). Additionally, cell cycle analysis revealed that co-treatment of DY and 5-FU resulted in a significant accumulation of cells in sub-G1 phase (Fig. 2c). In contrast to the HCT-116 cells, the RKO cells showed more necrosis but less apoptosis following DY treatment (Fig. 2b), and late apoptosis was predominantly responsible for the apoptosis observed in the RKO cells (Fig. 2c). Taken together, the apoptosis induced by DY may relieve defective apoptosis induced by 5-FU in both CRC cells.

### The combination of DY and 5-FU synergistically increased the ROS levels

**Figure 3:**
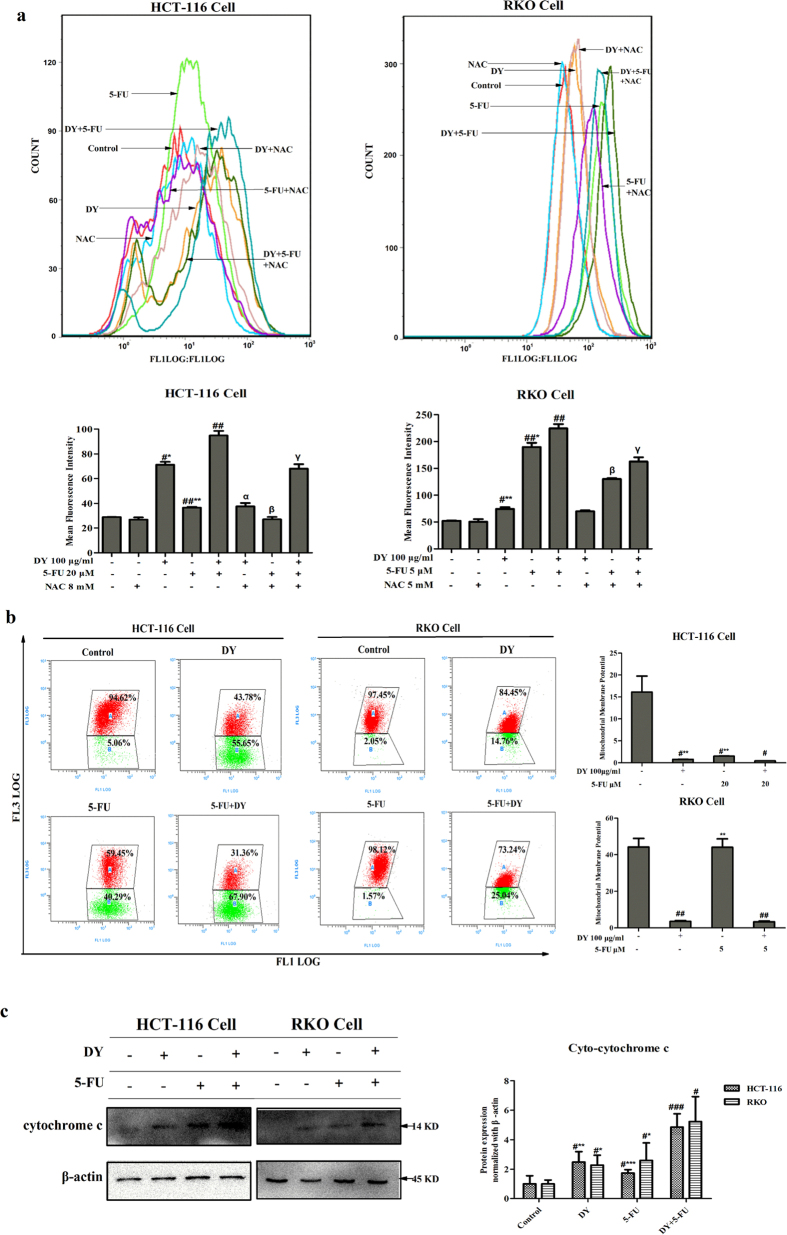
DY and 5-FU synergistically triggered the generation of ROS and disruption of MMP in CRC cells. (**a**) Cells were pre-treated with or without NAC, followed by DY and/or 5-FU. ROS levels of CRC cells loaded with DCFH-DA probes were evaluated by FCMS. (**b)** MMP levels were detected by JC-1 dye. JC-1 emits red fluorescence at high MMP levels by forming aggregate, while it emits green fluorescence as monomers at low MMP condition. The ratio of red/green fluorescence is used to indicate MMP levels. The distribution rates of J-aggregate and J-monomer in cells with DY/5-FU treatments are showed in the dot plots and cellular MMP levels are calculated in histograms. (**c**) After the indicated treatments, the cytosolic and mitochondrial fractions were isolated based on the manufacturer’s introduction of Cytochrome c Apoptosis Assay Kit. The expressions of cytosolic cytochrome c in two CRC cell lines were measured by western blotting analysis and normalized by β-actin, respectively. The levels of cytosolic cytochrome c were quantified in right histogram. The results are presented as mean ± SD (n = 3). ^**#**^P < 0.05, ^**##**^P < 0.01, ^**###**^P < 0.001, vs control group. While *P < 0.05, **P < 0.01, ***P < 0.001, vs (5-FU + DY) group. ^α^P < 0.05, ^β^P < 0.05 and ^γ^P < 0.05, vs the corresponding group without NAC pre-treatment.

Treatment with DY or 5-FU alone elevated ROS levels in both cell lines, and higher ROS levels were detected in the RKO cells. The combined treatment significantly increased the generation of ROS in both cell lines (Fig. 3a). When cells were pre-treated with NAC, the ROS levels of the cells following the single or combined treatments showed a significant decrease, except for a slight decline in the levels of RKO cells treated with DY. Annexin V-FITC/PI assays were conducted to evaluate the involvement of ROS in the synergistic apoptosis induced by DY and 5-FU. The results indicate that the induction of apoptosis was alleviated in cells pre-treated with NAC (see Supplementary Fig. S2a). Additionally, when ROS generation was suppressed by NAC, cell growth inhibition was partially abrogated in the DY and/or 5-FU group (Supplementary Fig. S2c). The results indicate that DY can synergistically trigger the generation of ROS in CRC cells when co-administered with 5-FU, which may be involved in the apoptosis induced by DY/5-FU.

### DY and 5-FU synergistically induced mitochondrial apoptotic pathway

Mitochondrial dysfunction can be induced by the elevation of ROS^19^, so we then evaluated whether DY and 5-FU can trigger mitochondrial disorders in a synergistic manner. The loss of mitochondrial membrane potential (MMP, *ΔΨm*), an indicator of mitochondrial inner membrane depolarization, was measured by JC-1 dye. Our results suggest that DY and 5-FU significantly triggered a loss of MMP, indicating mitochondrial depolarization may be induced, however, which was not observed in RKO cells (Fig. 3b).

The loss of MMP (mitochondrial depolarization) may result in the permeabilization of the mitochondrial outer membrane and activating apoptotic pathway by releasing apoptotic factors (cytochrome c, *et al.*) to cytoplasm^20^. Our data demonstrate that DY and 5-FU dramatically triggered a release of cytochrome c from mitochondrial outer membrane in HCT-116 cells, what’s interesting, in RKO cells as well (Fig. 3c and Supplementary Fig. S4a), indicating that the combined treatment permeabilized mitochondrial outer membranes and may induce mitochondria-dependent apoptotic pathway in a synergistic way in two CRC cell lines, which was confirmed in the following work.

### Mitochondrial dysfunction induced by DY and 5-FU contributed to caspase-dependent apoptosis

**Figure 4:**
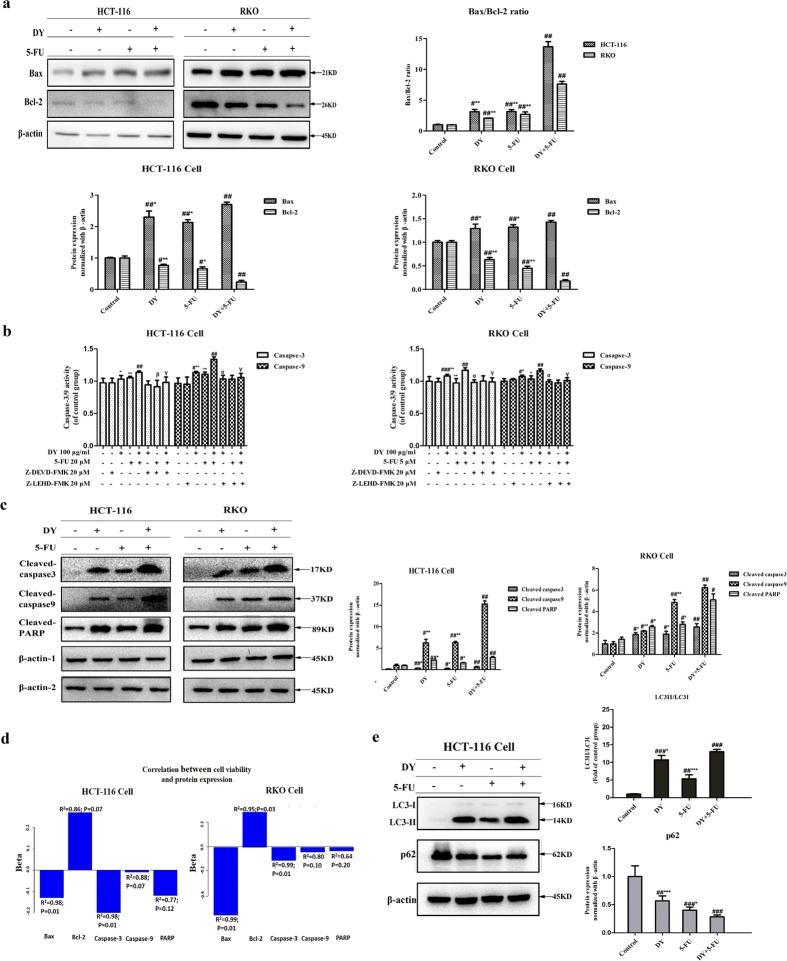
DY promoted 5-FU-induced apoptosis via a caspase-dependent apoptosis pathway. (**a**) Effects of 48 treatment with DY and/or 5-FU on the expression levels of Bax and Bcl-2 proteins in two CRC cell lines. (**b**) Activities of caspase-3/9 in two CRC cell lines treated with DY and/or 5-FU for 48 h with or without the caspase-3 & 9 inhibitors (Z-DEVD-FMK & Z-LEHD-FMK). (**c**) Expression levels of cleaved-caspase 3/9 and cleaved PARP in two CRC cell lines following the treatment with DY and/or 5-FU for 48 h were measured and normalized by β-actin (β-actin-1, cleaved caspase-3/9; β-actin-2, cleaved PARP). (**d**) Correlation between cell viability and protein expression was evaluated by a log-norm distribution analysis. (**e**) Cells were treated with either DY or 5-FU or their combination for 24 h, the expression levels of LC3I/II and p62 were then analyzed and respectively normalized by β-actin. The ratios of LC3II/LC3I and expression levels of p62 are presented in right histograms. All the values are expressed as mean ± SD (n = 3). ^#^P < 0.05, ^##^P < 0.01, ^###^P < 0.001, vs control group. While *P < 0.05, **P < 0.01, ***P < 0.001, vs (5-FU + DY) group, α, β and γ respectively indicated a significant difference was found between DY, 5-FU or DY + 5-FU group and their casapse-3/9 inhibition groups (P < 0.05).

Mitochondrial dysfunction is often involved in the mitochondrial/intrinsic apoptosis pathway. Bax and Bcl-2 play critical roles in regulating intrinsic apoptosis. In this study, we measured the expression levels of the pro-apoptotic protein Bax and anti-apoptotic protein Bcl-2. Notably, the combined treatment significantly increased the Bax protein, while decreased the Bcl-2 protein in both cell lines (Fig. 4a and Supplementary Fig. S4d). Based on a significant increase of Bax/Bcl-2 ratios, as well as the release of mitochondrial cytochrome c to cytosol, the caspase-dependent apoptosis pathway may be activated. So the activities of caspase-9 and caspase-3 were then evaluated. Data reveal that the initiator caspase-9, as well as its downstream executioner caspase-3 were activated in both cell lines following the combination treatments (Fig. 4b), which was also confirmed by western blot assays (Fig. 4c, Supplementary Fig. S4b,c). DY and 5-FU up-regulated the expression of cleaved caspase-9 and cleaved caspase-3 (P < 0.05). To clarify whether the activation of caspase-3/9 was associated with the apoptosis induced by DY and 5-FU, we further evaluated a downstream apoptotic indicator-poly (ADP-ribose) polymerase (PARP). Figure 4c shows that the cleaved PARP in CRC cells treated with DY and 5-FU was notably up-regulated (Supplementary Fig. S4e). Furthermore, when inhibiting the activities of caspase-3 and caspase-9 by Z-DEVD-FMK and L-LEHD-FMK, the apoptotic rates of the HCT-116 and RKO cells were reduced from 45.06 ± 1.44% to 36.44 ± 1.54% or 36.89 ± 0.92% and 12.24 ± 0.24% to 10.02 ± 0.42% or 10.26% ± 0.25%, respectively (Supplementary Fig. S2b, P < 0.05). Additionally, the anti-proliferative effects of the combined treatment were partially abrogated when cleavage of caspase-3/9 was blocked (Supplementary Fig. S2c). A univariate analysis showed that the five proteins all correlated with the viabilities of cells treated with DY and/or 5-FU (R^2^ > 0.6), and the correlations were stronger for Bax and caspase-3 (R^2^ > 0.9, P < 0.05) (Fig. 4d). Taken together, the results show that DY and 5-FU could cooperatively induce a ROS-mediated, mitochondria-caspase-dependent apoptosis, which probably accounts for their synergistic effects on the cytotoxicity in CRC cells.

### The roles of autophagy and HSP90 in the synergistic cytotoxicity induced by DY and 5-FU

For HCT-116 cells, a great number of cytoplasmic vacuoles were observed in DY or DY + 5-FU-treated groups (Fig. 1c), which actually emerged after 12 h-treatment and initially suspected to be induced by autophagy, so we then further investigated whether autophagy was involved in the synergistic death of HCT-116 cells. To achieve this, we evaluated two classic autophagy-related markers–light chain LC3 and p62 in HCT-116 cells with 24 h (a proper time-point for monitoring autophagy based on pre-experiments) treatments. During autophagy, LC3-I transforms to LC3-II while p62 is then degraded accordingly. The dramatic increase of the ratio of LC3-II/LC3-I along with a decline of p62 from Fig. 4c and Supplementary Fig. S4g indicate that DY indeed activated autophagy in HCT-116 cells and significantly enhanced 5-FU-induced autophagy, which might account for the synergistic cytotoxicity in HCT-116 cells (Fig. 1b, 24 h). In addition, we measured the expression levels of HSP90 in two CRC cell lines with the indicated treatments, however, which was not cooperatively inhibited by the combined treatments (Supplementary Figs S3 and S4f), indicating that HSP90 may be not involved in the synergism of DY.

### A combination of GA, CA and EA synergistically inhibited the proliferation of CRC cells with 5-FU

**Figure 5:**
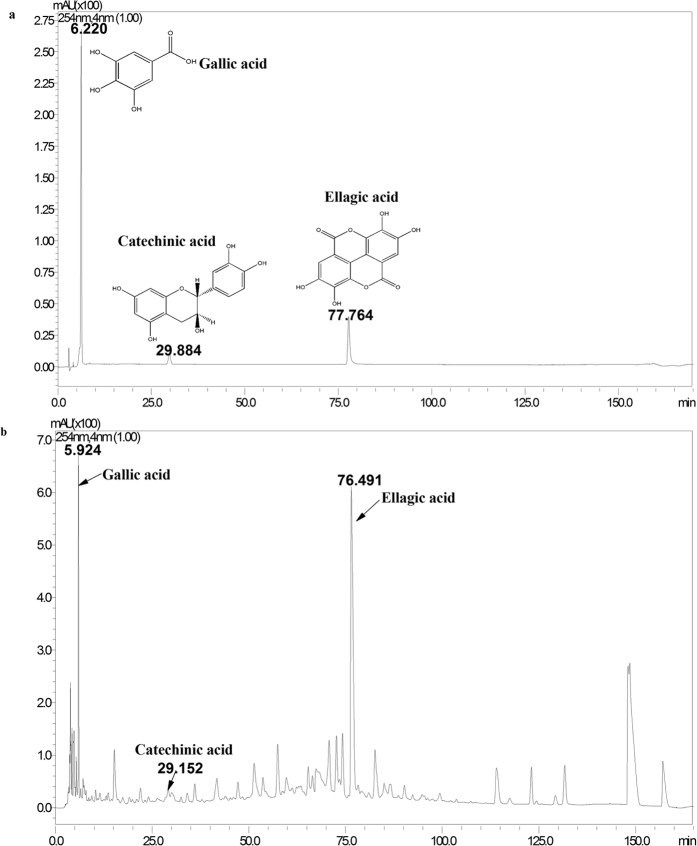
HPLC chromatograms of water extract of DY and its three tannins. (**a**) HPLC chromatograms of three tannins in DY (GA, CA and EA). (**b**) HPLC chromatograms of the overall extract of DY.

**Figure 6:**
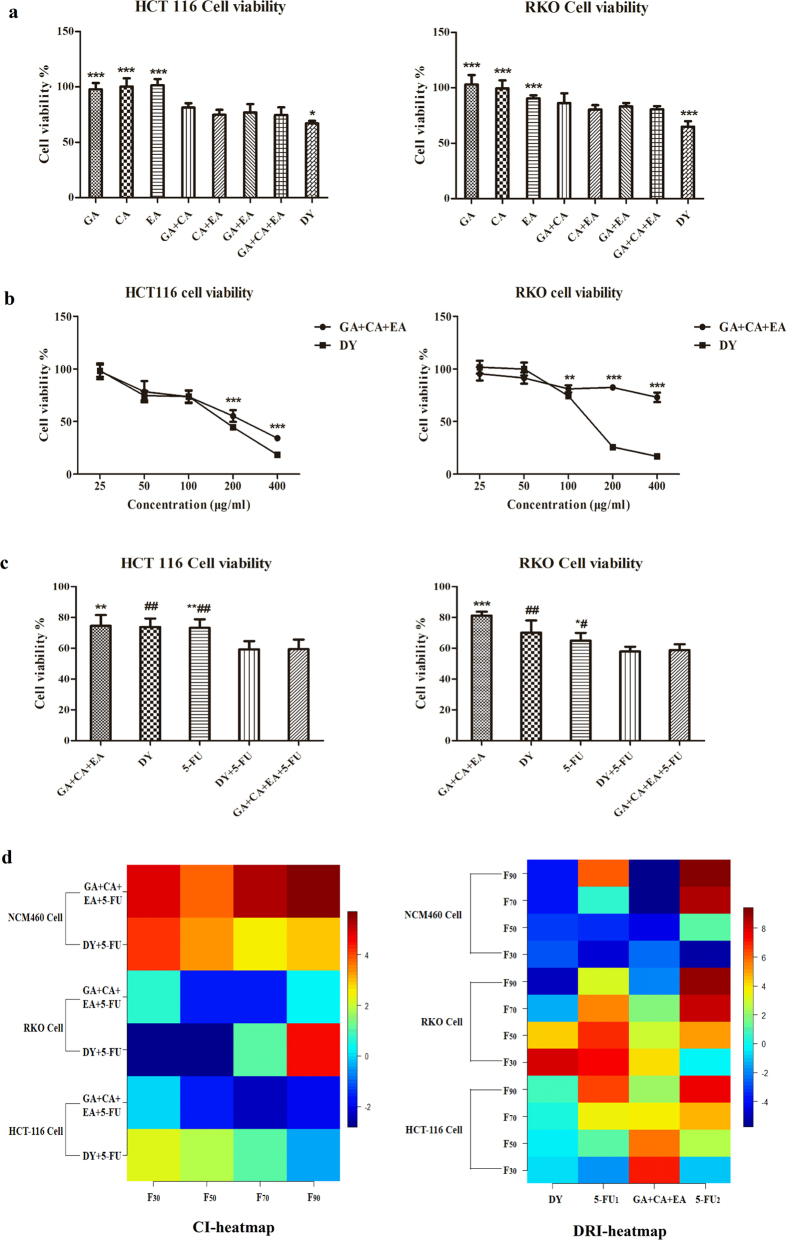
Three tannins in DY dramatically sensitized CRC cells to the 5-FU therapy. (**a**) Cells were treated with GA, CA or EA separately or in a combination, cell viabilities were evaluate by CCK8 assay. *P < 0.05, **P < 0.01, ***P < 0.001, compared with (GA + CA + EA) groups. (**b**) Concentration-effect curves of three tannins and DY were plotted, *P < 0.05, **P < 0.01, ***P < 0.001, vs DY groups. (**c**) The synergistic anti-proliferative effects of the three tannins and DY on the cytotoxicity of 5-FU in CRC cells. ^#^P < 0.05, ^##^P < 0.01, ^###^P < 0.001, vs (DY + 5-FU) group. While *P < 0.05, **P < 0.01, ***P < 0.001, vs (GA + CA + EA + 5-FU) group. All the data are expressed as mean ± SD (n = 3). (**d**) CI and DRI values from different combined groups are demonstrated in the heat-maps plotted by R programming, which are assessed by the color bars on the right (→red, values are greater; →blue, values are lower; 5-FU1 and 5-FU2 represent DRIs in the DY + 5-FU and tanins+5-FU groups, respectively; F_30_–F_90,_ 30% to 90% effective rate).

Tannins are the major components in DY (~17% weight of radix)^21^. Gallic acid (GA) and ellagic acid (EA) are representative hydrolysable tannins, while catechinic acid is one of the oligomeric procyanidins in DY. In this study, a water extract of DY was prepared using an extraction method with good reproducibility (RSD% of GA content = 1.39%, Supplementary Fig. 1a), and the three tannins in the overall extract of DY were separated and identified by HPLC analysis (Fig. 5 and Supplementary Fig. 1b). As determined by HPLC, the three tannins accounted for 3.9% of the total content of the DY extract. To investigate whether GA, CA and EA were the major bioactive constituents of DY that were responsible for the anti-proliferative effects on the CRC cell lines, we evaluated the cytotoxicity of each tannin in CRC cells. No obvious inhibitory activity was observed for any of the tannins (IC_50_ > 100 μM). Then, we concurrently treated the cells with two or three tannins at same concentrations in 100 μg/ml DY extracts. The cell viabilities were significantly inhibited by the combined tannins (P < 0.05, vs single tannin group), and treatments with two or three tannins showed comparable potency in inhibiting the CRC cells (we chose the combination of three tannins for further research considering its better cooperation). However, the inhibitory effects were lower than that of the overall DY extract (P < 0.05) (Fig. 6a). The dose-effect curves of the three tannins are shown in Fig. 6b. The HCT-116 cells were more sensitive to the tannin treatment than the RKO cells, the growth of which was notably inhibited in a dose-dependent manner. The inhibitory effects of the tannins and the DY extract were comparable at low concentrations (25–50 μg/ml). However, with increased concentrations, the anti-proliferative effects of the tannins turned to be much lower than that of the DY treatment. These results indicate that GA, CA and EA together could effectively inhibit the CRC cells but can’t replace DY.

We found that DY showed synergistic effects to 5-FU by inhibiting HCT-116 and RKO cell viabilities, and co-treatment with GA, CA and EA also exhibited cytotoxicity against CRC cells. Thus, we determined to evaluate whether the three tannins could synergistically enhance the cytotoxicity of 5-FU. CCK8 assays indicated that the synergistic effects of tannins towards 5-FU cytotoxicity was comparable to the DY extract at the same concentration (Fig. 6c), which was also confirmed by the CI and DRI values calculated by the synergistic formula (Table 1 and Fig. 6d), indicating that the three tannins are likely to be the primary components that account for the synergism of DY in 5-FU cytotoxicity.

## Discussion and Conclusion

An increasing number of studies have indicated that *Sanguisorba officinalis* L. radix (Rosaceae) exerts potent anti-cancer activities against several types of tumours, such as breast cancer, prostate cancer or oral cancer^8^,^9^,^10^. However, the efficiency of DY in treating CRC has not yet been reported. Tannins, the major constituents of DY, exhibit anti-cancer effects towards CRC in many natural plants (such as green tea, strawberry and grapes)^22^,^23^,^24^; therefore, we hypothesized that DY may suppress the development of CRC, and tannins likely played an important role in the anti-cancer activity. This study first assessed the anti-CRC efficiency of DY, and the major components responsible for these effects were identified as well. Our data indicate that a water extract of DY could inhibit the proliferation of HCT-116 and RKO cells at a relatively low dose (IC_50_ < 200 μg/ml)^25^, while it exerted only a slight cytotoxicity towards normal cells (Fig. 1a). Subsequently, the cytotoxicity of the three major tannins (gallic acid, catechinic acid and ellagic acid) of DY was evaluated. Data show that the combination of the three tannins only exhibited comparable efficiency to DY at low doses; the anti-proliferative activity of the tannins was significantly inferior to the overall DY extract at high doses (Fig. 6b), indicating that other constituents may cooperatively contribute to the efficiency of DY.

5-FU is a first-line chemotherapeutic agent for CRC, but its application is hampered by drug resistance and toxicity at high doses^26^. To achieve better clinical effects, 5-FU is often used in combination regimens with other drugs^27^,^28^,^29^. Our study found that DY can synergistically enhance the anti-proliferative effects of 5-FU against CRC cells (Fig. 1b). As dysfunctional apoptosis partially accounted for the failure of 5-FU therapy in CRC^4^, we then studied whether apoptosis was involved in the synergism of the combined treatment. Data reveal that apoptosis was dramatically induced by DY and 5-FU treatment in the two cell lines (Fig. 2). And further investigation suggested that the synergistic apoptosis may be induced by the ROS-mediated, mitochondria-caspase-dependent apoptotic pathway. DY synergistically triggered ROS generation, up-regulated Bax/Bcl-2 ratios (an indicator for cells undergoing apoptosis^30^) and disrupted mitochondrial membrane in CRC cells treated with 5-FU (Fig. 3a,b), resulting in the release of cytochrome c to the cytosol (Fig. 3c). Additionally, co-treatment of DY and 5-FU dramatically activated caspase-9 and caspase-3 activities (Fig. 4a,b). The elevation of cleaved PARP (a marker of apoptosis^31^) was also detected in CRC cells treated with the combined regimen (Fig. 4c). All the above effects were blocked by inhibitors of ROS and caspase-3/9 (Supplementary Fig. S2), indicating that the intrinsic apoptotic pathway is involved in the synergism of DY and 5-FU^6^, which has also been found in cancer cells treated with 5-FU and other herbs, such as Asian ginseng^32^ or the American ginseng berry^33^. Univariate analyses are generally used to predict prognostic markers for cancer therapy^34^. Therefore, in this study, we assessed the correlation of the five apoptotic proteins with cytotoxicity of DY and/or 5-FU by a Gaussian Distribution Function and the results indicate that the expressions of Bax and caspase-3 showed strong negative correlations with CRC cell viability in both cell lines (Fig. 4d). Thus, we hypothesized that DY may sensitize CRC cells to 5-FU therapy predominantly by enforcing the expression of the two proteins. However, because the sample size is small, this hypothesis should be confirmed in future studies.

In addition to the synergistic induction of apoptosis, activating autophagy and blocking heat shock protein 90 are also reported to sensitize CRC cells to chemotherapy^35^,^36^. Based on the western blotting analysis of two autophagic markers-LC3 and p62^37^, our study verified that DY activated autophagy in HCT-116 cells at 24 h, which even synergistically promoted the 5-FU-induced autophagy (Fig. 4e). Previous studies proposed that agents that can induce autophagy or autophagic death in tumour cells may be promising anti-cancer therapeutics^38^, and activators of autophagy might be able to sensitize cancer cells towards chemotherapeutics^39^. So we hypothesized that, the enhanced autophagy induced by DY and 5-FU might account for the synergism of DY in HCT-116 cells at 24 h (Fig. 1b). In addition, increasing evident show that HSP90 has become a promising anticancer target and HSP90 inhibitors have exerted anticancer activities towards colon cells^39^. However, though DY inhibited HSP90 expression in both CRC cell lines, the synergistic down-regulations of HSP90 were not observed in our study (supplementary Fig. S3), implying that HSP 90 might be not involved in the synergism of DY and 5-FU.

Apoptosis plays an important role in the mechanism of cytotoxicity of DY, as well as the synergistic anti-proliferative effects of DY and 5-FU, but we found that apoptosis induced by DY in RKO cells was not as apparent as in the HCT-116 cells, which is consistent with Thant *et al.*’s findings^40^. In addition to apoptotic cells, a large number of necrotic cells were also found in RKO cells treated with DY or DY + 5-FU (Fig. 2b), indicating that other patterns of programmed cell death (necrosis) might be involved in the cytotoxicity of DY in RKO cells. Figure 3 shows that DY dramatically increased the generation of ROS and disrupted the mitochondria. Previous reports have suggested that excess ROS and mitochondrial dysfunction could induce cellular necrosis^41^. Therefore, DY-mediated necrosis in RKO cells may be due to ROS/mitochondrial dysfunction. Additionally, according to our data, mitochondrial dysfunction was involved in synergistic apoptosis in RKO cells, which was mainly contributed by the permeabilization of outer membrane (Fig. 3c). Evidences clarify that mitochondrial inner membrane depolarization can rupture outer membrane to induce a release of apoptotic factors^20^, however, which was not observed in RKO cells just like in HCT-116 cells (Fig. 3b). Recent studies indicated that the improvement of mitochondrial permeabilization can also be mediated by opening a mitochondrial apoptosis-induced channel (MAC) in the outer membrane, which is regulated by Bcl-2 family proteins without mitochondrial depolarization^20^,^42^, so we guess that the mitochondrial disruption in RKO cells may be mitochondrial depolarization-independent but mainly be due to an open of MAC. The similar results are also found in Priault *et al.*’s study^43^.

Although the anti-proliferative abilities of GA, CA and EA against CRC have been shown in many reports^44^,^45^,^46^, our study found that the combination of the three tannins cannot be used as a replacement of the overall extract of DY in CRC therapy (Fig. 6b). However, interestingly, we found that satisfactory synergistic anti-proliferative effects against CRC cells were achieved by the three tannins, along with an antagonistic effect on the cytotoxicity of 5-FU to the normal colon cells (Fig. 6d and Table 1). Several studies reported that tannins were able to sensitize cholangiocarcinoma cells to the cytotoxicity of chemotherapeutic agents^47^, indicating that the three tannins in DY are likely to account for the synergistic effects of DY on 5-FU therapy. However, this hypothesis, as well as the underlying mechanism and the optimized combination of the three tannins, should be verified and studied in future work.

**Figure 7:**
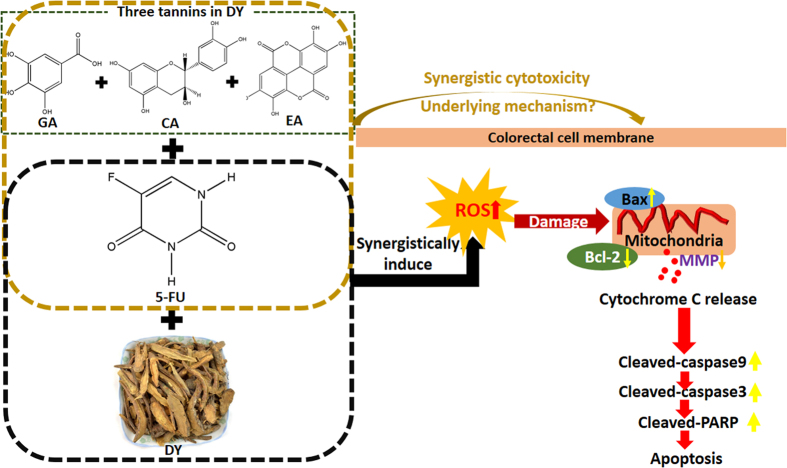
A schematic diagram of a ROS-mediated, mitochondria-caspase-dependent apoptosis involved in mediating the synergistic anti-proliferative effects of DY/tannins and 5-FU against CRC. DY and 5-FU synergistically promoted the generation of ROS in CRC cells and triggered a mitochondria disruption (cytochrome c release) by boosting the ratio of Bax/Bcl-2, which then activated caspase-9/3 and PARP. A combination of GA, CA and EA exhibited comparable synergistic effects on 5-FU cytotoxicity to DY water extract, but the underlying mechanism needs to be explored.

In conclusion, a water extract of DY was first found to have anti-proliferative activity against CRC cells. In addition, DY sensitized CRC cells to 5-FU therapy and alleviated the cytotoxicity of 5-FU on normal cells. A mechanistic study revealed that a ROS-mediated, mitochondria-caspase-dependent apoptotic pathway is possibly involved in the synergism. For HCT-116 cells, autophagy induced by DY may also help to enhance the cytotoxicity of 5-FU. Moreover, a combination of three tannins in DY, namely GA, CA and EA, was comparable to the overall DY extract in mediating the synergistic cytotoxicity with 5-FU, suggesting a promising combination therapy strategy for 5-FU-resistant CRC. These findings (Fig. 7) should be confirmed in drug-resistant CRC cells and, most importantly, in vivo; these studies are currently in progress.

## Materials and Methods

### Plant materials

*Sanguisorba officinalis L.* radix (Jiangsu, China; Batch Number-20150302) was purchased from Zhixin Medicine Health Co., Ltd. (Guangzhou, China) and was authenticated by Prof. Depo Yang, Sun Yat-sen University. The moisture content of the sample was 8.11 ± 0.48%. We preserved the voucher specimens (Voucher Number: DY-JS-20150305) in the Lab of Pharmaceutical Analysis and Quality Assessment, School of Pharmaceutical Sciences, Sun Yat-sen University, China.

### Chemicals and reagents

We obtained 5-fluorouracil (99%) from the Zhenzhou Huawen Chemical Co., Ltd. (Zhenzhou, China). Gallic acid (<98%), catechinic acid (98%) and ellagic acid (98%) were purchased from Chengdu PUSH Bio-technology Co., Ltd. (Chengdu, China). The CCK8 kit was from Dojindo (Kumamoto, Japan). The Hoechst 33258 staining solution, the mitochondrial membrane potential assay kit with JC-1, the Reaction Oxygen Species Assay Kit and N-acetyl-L-cysteine (NAC) were purchased from Beyotime (Jiangsu, China). The Annexin V-FITC Apoptosis Detection kit and the PI/RNase Staining Buffer were obtained from BD Biosciences (New Jersey, USA). The Caspase-3 and Caspase-9 Colorimetric Assay Kit, together with Z-DEVD-FMK, Z-LEHD-FMK and Cytochrome c Apoptosis Assay Kit were purchased from Biovision, Inc. (Milpitas, CA). The primary antibodies against Bcl-2 and the secondary antibody were obtained from EMD Millipore Corporation (Temecula, CA). Anti-Bax was from Abcam (Cambrige, MA, USA) and anti-β-actin and anti-HSP 90 were from Cell Signaling Technology (Beverly, MA, USA). Anti-cleaved caspse-3/9 and anti-cleaved PARP, and anti-LC3B and anti-p62 were a kind gift from Dr. Hongsheng Wang and Dr. Min Li (Sun Yat-sen University), respectively.

### Plant extract preparation and HPLC analysis

*Sanguisorba officinalis* L. radix (10 g) was ground to 40 mesh pieces and boiled at 80–85 °C in hot water for 1 h. Then, the extract was filtered to obtain the supernatant. The residue was repeatedly extracted twice. Then, the total filtrate was combined and concentrated using a rotary evaporator at 0.09 MPa (65 °C). The concentrate was stored at −80 °C overnight and lyophilized for 24 h to obtained raw powder for further use. The yield was 42% for the DY extract. To obtain the chemical profile of the DY extract, a high-performance liquid chromatography (HPLC) method was developed. Before HPLC analysis, the extract powder was dissolved in distilled water and extracted for 30 min by ultrasound, and the reference substances (GA, CA, EA) were prepared in purified water or methanol and then filtered through a 0.22 μm membrane to acquire the pre-run samples. The HPLC analysis was conducted on a Luna (C18) 100A column (250 mm × 4.6 mm, 5 μm) (Phenomenex, CA, USA). The LC analysis was carried out as described by Wang *et al.*^8^ with minor changes. In brief, the mobile phase was comprised of 0.2% formic acid (A) and acetonitrile (B), and the elution programme was as follows (B%): 10 min, 5%; 80 min, 15%; 130 min, 25%; 150 min, 45%; 155 min, 80%; 160 min, 5%; 170 min, 5%. To guarantee the reproducibility of this extraction method, the relative standard deviations (RSD) of the content percentages of gallic acid (a representative constituent in DY^48^) were evaluated. A good reproducibility is achieved when RSD% < 5%.

### Cell lines and culture

The human NCM 460, HCT-116 and RKO cell lines were a kind gift from Prof. Jun Du (Sun Yat-sen University). All the cell lines were cultured in DMEM medium supplemented with 10% foetal bovine serum and 1% penicillin-streptomycin (Gibco, China), except for the HCT-116 cell line, which was maintained in RPMI 1640 medium (Gibco, China). Cells were incubated in a 5% CO_2_ and 37 °C atmosphere.

### Cell viability assay

Cells were seeded in 96-well plates at a density of 10^4^ cells per well. After a 12 h wait for attachment, the culture medium was replaced with different drug solutions (DY, a combination of GA, CA and EA at the same concentration as in 100 μg/ml DY^49^, or 5-FU). After 24 or 48 h, CCK-8 assays were performed to evaluate the cell viability.

### Synergistic experiments

To determine the CI and DRI values for DY/tannins (GA + CA + EA) and 5-FU, 0.125, 0.25, 0.625, 1.25, 2.5 times the IC_50_ of the DY/tannins combination and 5-FU were concurrently added to CRC and normal cells, and 48 h later, cell viabilities were evaluated by CCK8 assays. The data were analysed using CompuSyn software (Biosoft, Ferguson, MO, USA), which is based on the drug combination theory of Chou^17^, to calculate the CI and DRI values. CI < 1, CI = 1 and CI > 1 are considered synergistic, additive and antagonistic effects, respectively.

### Crystal violet staining assay

HCT-116 and RKO cells were seeded in 6-well plates (4 × 10^5^ cells/well), and after attachment, the cells were treated with DY (100 μg/ml) and/or 5-FU (20 μM, HCT-116; 5 μM, RKO). After 48 h exposure to drugs, the cells were washed twice with PBS and fixed in 4% paraformaldehyde (Boster, Wuhan, China) for 10 min. Subsequently, the cells were washed and stained with crystal violet staining solution for 10 min. Cellular morphologies were then photographed under an inverted microscope (Olympus, Tokyo, Japan).

### Hoechst 33258 staining assay

After 48 h drug exposure, cells were fixed with 4% paraformaldehyde for 30 min before staining with Hoechst 33258 solution. The morphological changes in the cells were photographed by an inverted fluorescence microscope (Olympus, Tokyo, Japan). Cells with condensed and bright/fragmentary nuclei are considered to undergo apoptotic. Three visual fields (100 cells/field) were chosen to calculate the apoptotic rates in each group. Mean fluorescence intensity in single cell was calculated by the High Content Screening System (ArrayScanVTI, Thermo Fisher, USA).

### Flow cytometry analysis of apoptosis and the cell cycle

Cells were collected and washed twice with cold PBS, and they were then prepared according to the protocol of the Annexin V-FITC Apoptosis Detection Kit. In cell cycle assays, the cells were first synchronized by serum starvation for 24 h. After drug treatment (48 h), the cells were washed with ice-cold PBS and dispersed before adding pre-cooled 70% ethanol (stored at −20 °C overnight). Then, cells were washed with PBS and incubated with 500 μL PI/RNase solution for 15 min. The prepared cellular samples were immediately analysed on a Coulter Epics XL Flow Cytometric System and processed by Summit 5.2 (Beckman Coulter, Miami, FL, USA).

### Reactive oxygen species assay (ROS)

DCFH-DA reagent was used to detect the reactive oxygen species (ROS) levels in CRC cells. Cells were pre-treated with or without 8 mM (HCT-116) or 5 mM (RKO) N-acetyl-L-cysteine (NAC) for 30 min followed by different treatments. After 48 h, the cells were collected, 5 μM (HCT-116 cells) and 10 μM (RKO cells) DCFH-DA probes were added, and the cells were incubated at 37 °C for 40 min. Then, fluorescence excitation at 488 nm and emission at 525 nm were monitored using a flow cytometry system (FCMS). In addition, Annexin V-FITC/PI and CCK-8 assays were conducted to evaluate the effects of ROS on apoptosis and cell death induced by DY and/or 5-FU.

### Measurement of mitochondrial membrane potential (MMP)

The decrease in mitochondrial membrane potential (MMP, *ΔΨm*) is a marker of early apoptosis in living cells. We collected cells 48 h after treatment and incubated them with a JC-1 (5,5′,6,6′,-tetrachloro-1,1′,3,3′-tetraethyl benzimidazolyl-carbocyanine iodide) working solution for 20 min at 37 °C. Cells were then washed twice and resuspended in JC-1 buffer (1×), followed by analysis of JC-1 fluorescence using FCMS.

### Western blot analysis

The cellular lysates were collected. The concentrations of total proteins were determined using the bicinchoninic acid (BCA) method. Then, 30 μg of each cellular sample was loaded in an 8–15% gel and subject to sodium dodecyl sulphate-polyacrylamide gel electrophoresis (SDS-PAGE). The gels were transferred to PVDF films (Millipore, Darmstadt, Germany), blocked for 2 h and blotted with the indicated antibodies at room temperature for 2 h. After washing three times (5 min for each time) with 1× TBS, the films were cultured with HRP-conjugated anti-rabbit secondary antibody for 1 h. Then, SuperSignal^TM^ West Pico Chemiluminescent Substrate (Thermo Fisher Scientific, MA, USA) was used to visualize the protein bands, which were exposed and imaged with a ChemiDoc XRS+ system (Bio-Rad, Hercules, CA, USA).

### Caspase 3/9 activity assays

Caspase-9/3 activities in CRC cells pre-treated with/without 20 μM Z-DEVD-FMK (caspase-3 inhibitor) or L-LEHD-FMK (caspase-9 inhibitor) were detected following the manufacturer’s instructions from the Caspase Colorimetric assay kits. 100 μg protein was incubated with 200 μM DEVD-pNA substrate (caspase-3) or LEHD-pNA substrate (capase-9) at 37 °C for 2 h. Then, the absorbance of the samples was read at 400 nm by a microplate reader (Molecular Devices, CA, USA). In addition, apoptosis rates and cellular viabilities of two cell lines with the above treatments were analysed.

### Statistical analysis

All the data were obtained from three repeated experiments and were expressed as the mean 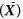  ± SD. Two-tailed Student’s t-test was used with SPSS 18.0 software (SPSS Inc., Chicago, IL, USA) to conduct the statistical analysis. P < 0.05 indicated a significant difference. R programming was used to plot the heat maps, as well as to conduct the univariate analysis using a Gaussian Distribution (R^2^, correlation coefficient; Beta, clarifying positive or negative correlation).

## Additional Information

**How to cite this article**: Liu, M. P. *et al.* *Sanguisorba officinalis* L synergistically enhanced 5-fluorouracil cytotoxicity in colorectal cancer cells by promoting a reactive oxygen species-mediated, mitochondria-caspase-dependent apoptotic pathway. *Sci. Rep.* **6**, 34245; doi: 10.1038/srep34245 (2016).

## Supplementary Material

Supplementary Information

## Figures and Tables

**Table 1:** Synergistic analysis of DY/Tannins and 5-FU cytotoxicity on neoplastic and normal colon cells.

Combination groups	Cell lines	Parameters	F_30_	F_50_	F_70_	F_90_
**DY** + **5-FU**	HCT-116	CI	1.11	0.87	0.72	0.57
		DRI(DY)	1.99	2.06	2.13	2.25
		DRI(5-FU)	1.66	2.59	4.04	8.22
	RKO	CI	0.15	0.31	0.72	3.3
		DRI(DY)	14.60	5.08	1.77	0.33
		DRI(5-FU)	11.79	8.73	6.47	4.01
	NCM460	CI	3.15	2.04	1.73	1.99
		DRI(DY)	1.28	1.00	0.79	0.54
		DRI(5-FU)	0.42	0.96	2.20	8.21
Tannins + 5-FU	HCT-116	CI	0.63	0.45	0.38	0.41
		DRI(Tannins)	8.75	6.50	4.83	3.01
		DRI(5-FU)	1.93	3.32	5.73	13.64
	RKO	CI	0.68	0.45	0.45	0.66
		DRI(Tannins)	4.96	3.59	2.60	1.55
		DRI(5-FU)	2.09	5.82	16.21	83.04
	NCM460	CI	3.90	2.71	7.61	49.72
		DRI(Tannins)	1.41	0.43	0.13	0.02
		DRI(5-FU)	0.31	2.59	21.47	623.04

Cells were treated with DY or Tannins (GA + CA + EA), with or without 5-FU for 48 h. CI and DRI values were calculated by Compusyn software. Fa, effect levels. Fa_30_–Fa_90_, 30–90% efficient percentage. CI means combination Index, CI = (D)_1_/(D_x_)_1_ + (D)_2_/(D_x_)_2_, (D_x_)_1_ or (D_x_)_2_ represents the dose of drug 1 or 2 in a combination needed for achieving the same efficiency as that of the single drug 1 or 2 at D_1_ or D_2_, respectively. And CI < 1, CI = 1 and CI > 1 indicates synergistic, additive and antagonistic effects, respectively. Dose reduction index (DRI) indicates the folds of dose for each drug to be reduced in a combination compared to a single drug at a given effect. The greater of DRI values, the more dose of a single drug would be reduced to achieve the same effect. Both of DRI (DY)/(Tannins) and DRI (5-FU) that are greater than one would result in a synergistic effects for the combined treatment.
