# Supplementary material for: Sanguisorba officinalis L synergistically enhanced 5-fluorouracil cytotoxicity in colorectal cancer cells by promoting a reactive oxygen species-mediated, mitochondria-caspase-dependent apoptotic pathway
Source: Sci Rep. 2016 Sep 27;6:34245. doi: 10.1038/srep34245 (PMC5037464; doi:10.1038/srep34245)
Supplement: Supplementary Information [file srep34245-s1.pdf]

Sanguisorba officinalis L synergistically enhanced 5-fluorouracil cytotoxicity in colorectal cancer cells by promoting a reactive oxygen species-mediated, mitochondria-caspase-dependent apoptotic pathway

Meng-ping Liu[1,§], Min Liao[1,§], Cong Dai[1], Jie-feng Chen[1], Chun-juan Yang[2], Ming Liu[1], Zuan-guang Chen[1] and Mei-cun Yao[1,*]

[1]School of Pharmaceutical Sciences, Sun Yat-sen University, Guangzhou 510006, P.R. China.

[2]College of Pharmacy, Harbin Medical University, Harbin 150081, P.R. China.

[§]The authors contributed equally to this work.

*Corresponding author. Correspondence and requests for materials should be addressed to M.C.Y. (E-mail: yaomeicun@gmail.com)

The email addresses of the above author are listed as follows,

M.P.L: liump3@mail2.sysu.edu.cn

M.L: mnliao@126.com

C.D: 1533636644@qq.com

J.F.C: 794615587@qq.com

C.J.Y: chunjuanyang@126.com

M.L: 303973282@qq.com

Z.G.C: chenzg@mail.sysu.edu.cn.

Supplementary figure S1

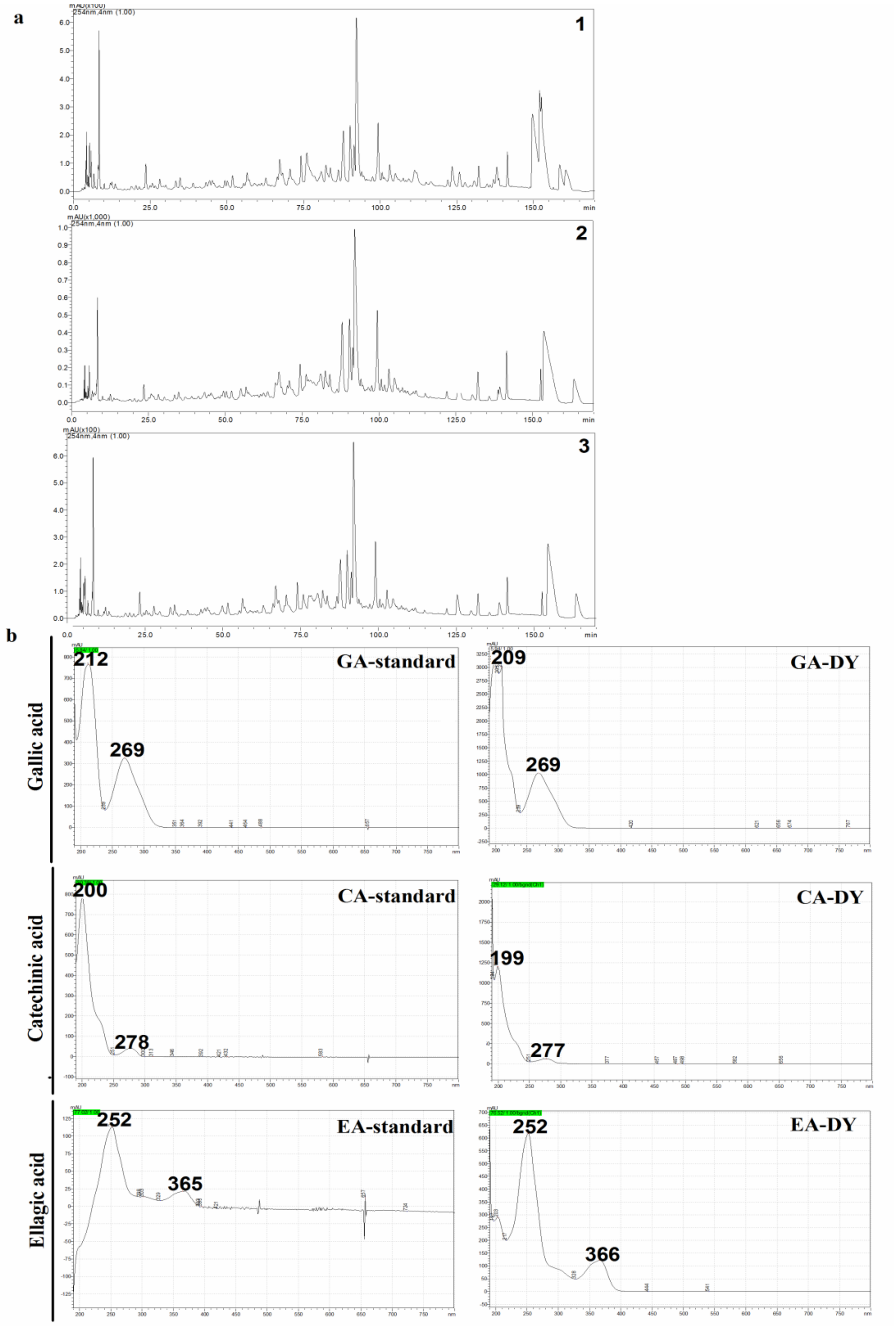

Supplementary Figure S1. **HPLC chromatograms of DY water extracts from three independent extraction experiments.** (**a**) The chromatograms of DY extracted from 4.97 (1), 5.12 (2) and 4.92 g (3) crud drugs, respectively. (**b**) The UV spectrograms of GA, CA and EA in standard solutions or the overall extracts of DY.

Supplementary figure S2

a

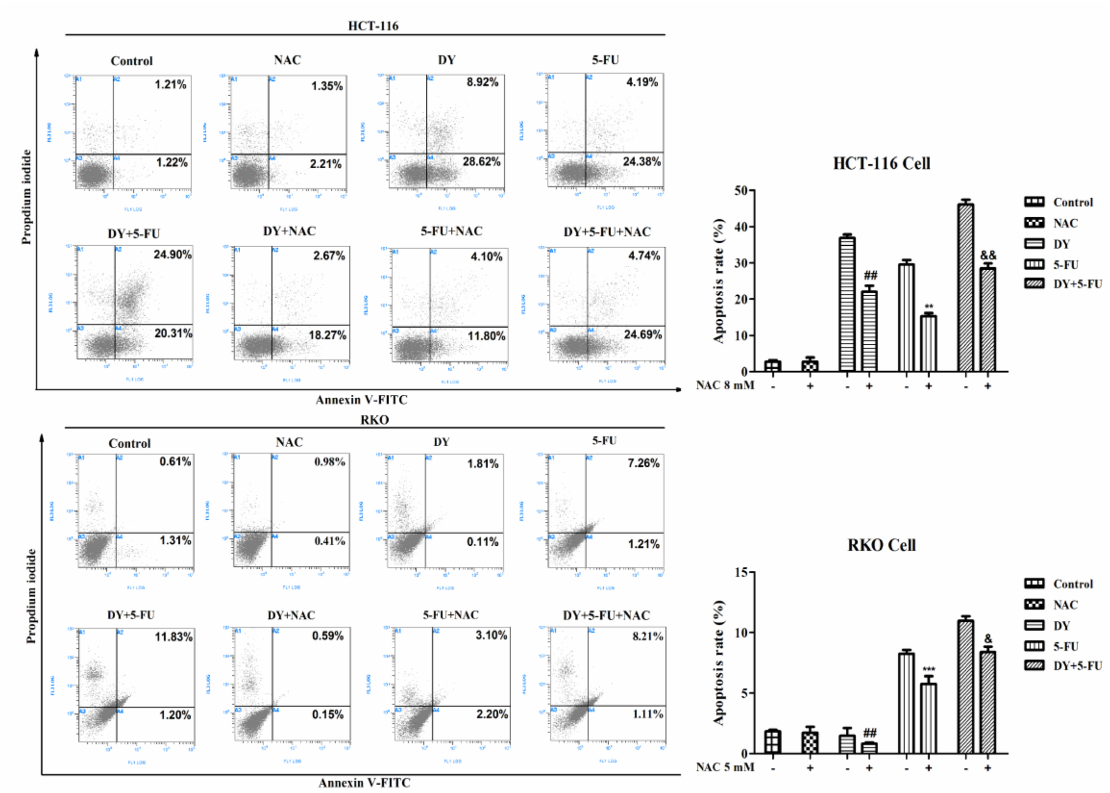

b

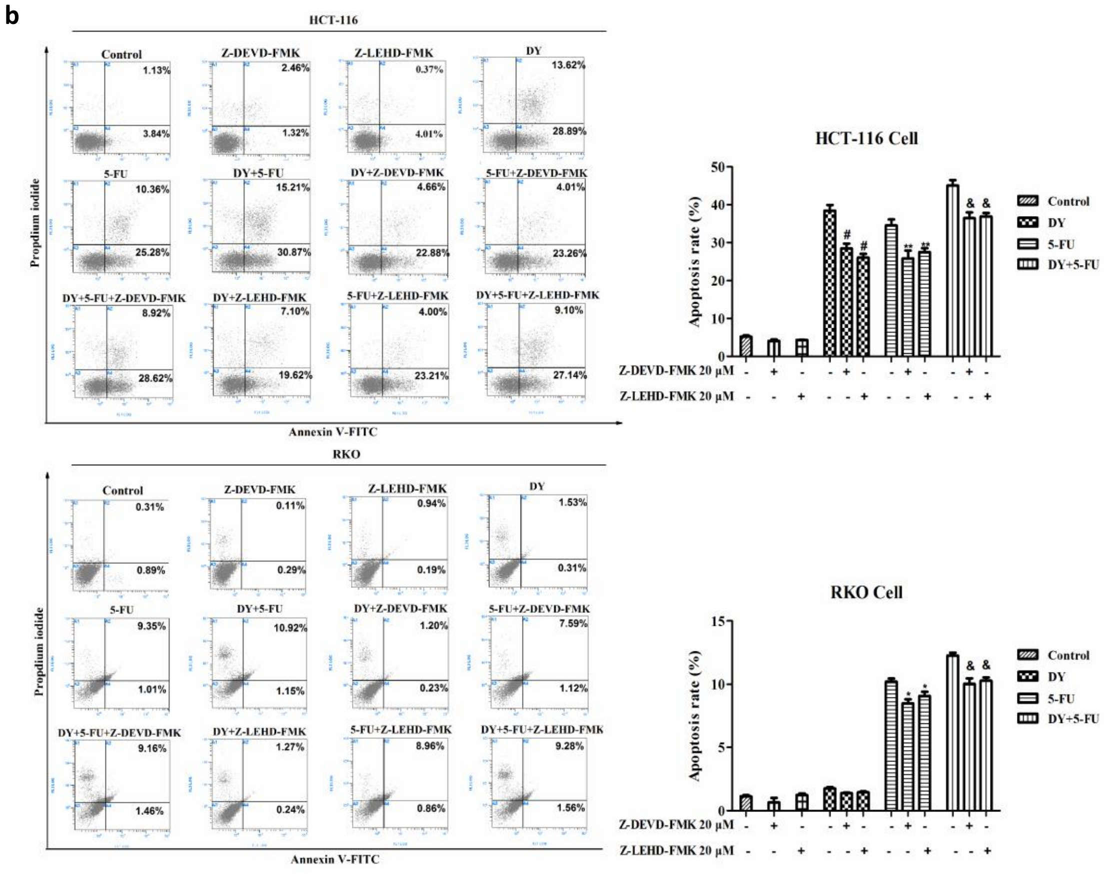

c

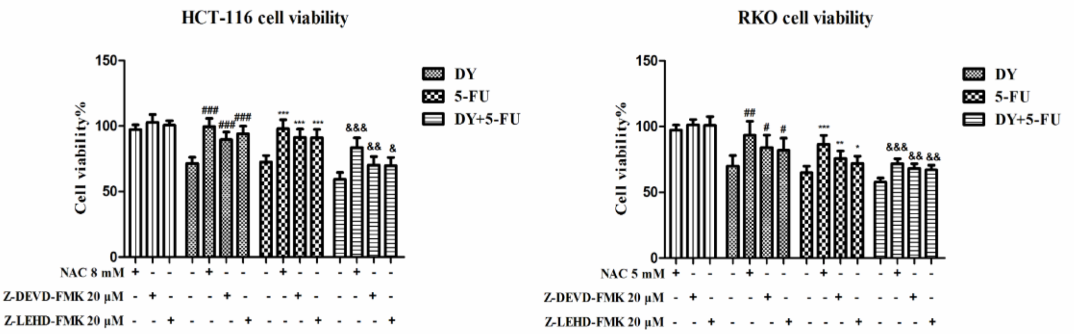

Supplementary Figure S2. A ROS-mediated, and caspase-3/9-dependent apoptosis pathway was involved in the synergistic anti-proliferation effects of DY and 5-FU in CRC cells. (**a**) & (**b**) CRC cells were pre-treated with or without ROS inhibitor NAC, caspase-3 inhibitor Z-DEVE-FMK or caspase-9 inhibitor Z-LEHD-FMK, followed by DY and/or 5-FU for 48 h. Apoptosis rates were measured by FCMS, while cell viability was assessed by CCK8 assay (**c**). Data is all presented as mean ± SD (n=3). $^{\#}P<0.05$, $^{\#\#}P<0.01$, $^{\#\#\#}P<0.001$, vs DY group. *P<0.05, **P<0.01, ***P<0.001, compared with 5-FU group. While $^{\&}P<0.05$, $^{\&\&}P<0.01$, $^{\&\&\&}P<0.001$, vs (DY+5-FU) group.

Supplementary Figure S3

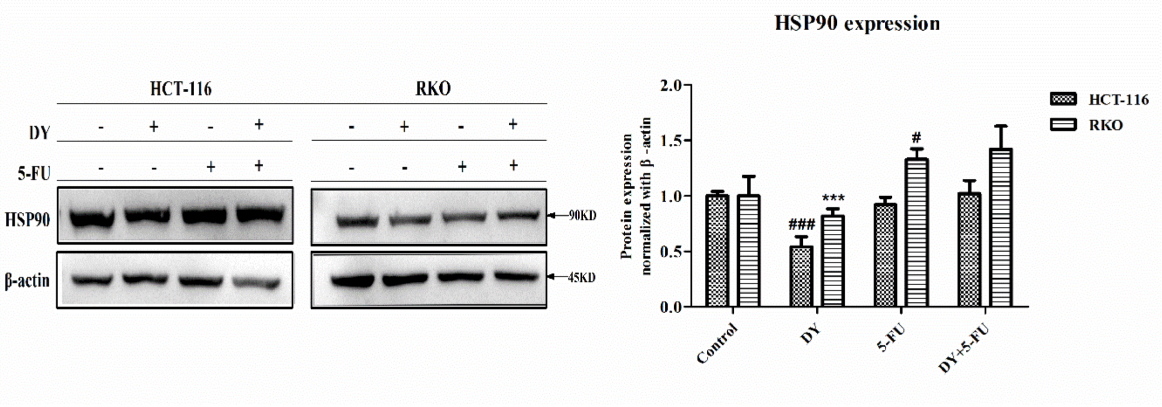

Supplementary Figure S3. The combination of DY and 5-FU might not be able to synergistically inhibit the expression of HSP90 in CRC cells. The expression levels of HSP90 in CRC cells treated with DY/5-FU alone, or in a combination were evaluated by western blotting and normalized by β-actin. Relative expression levels of HSP 90 are showed as mean ± SD (n=3), $^{\#}P<0.05$, $^{\#\#\#}P<0.001$, vs control group. ****P<0.001, vs DY+5-FU group.

Supplementary Figure S4

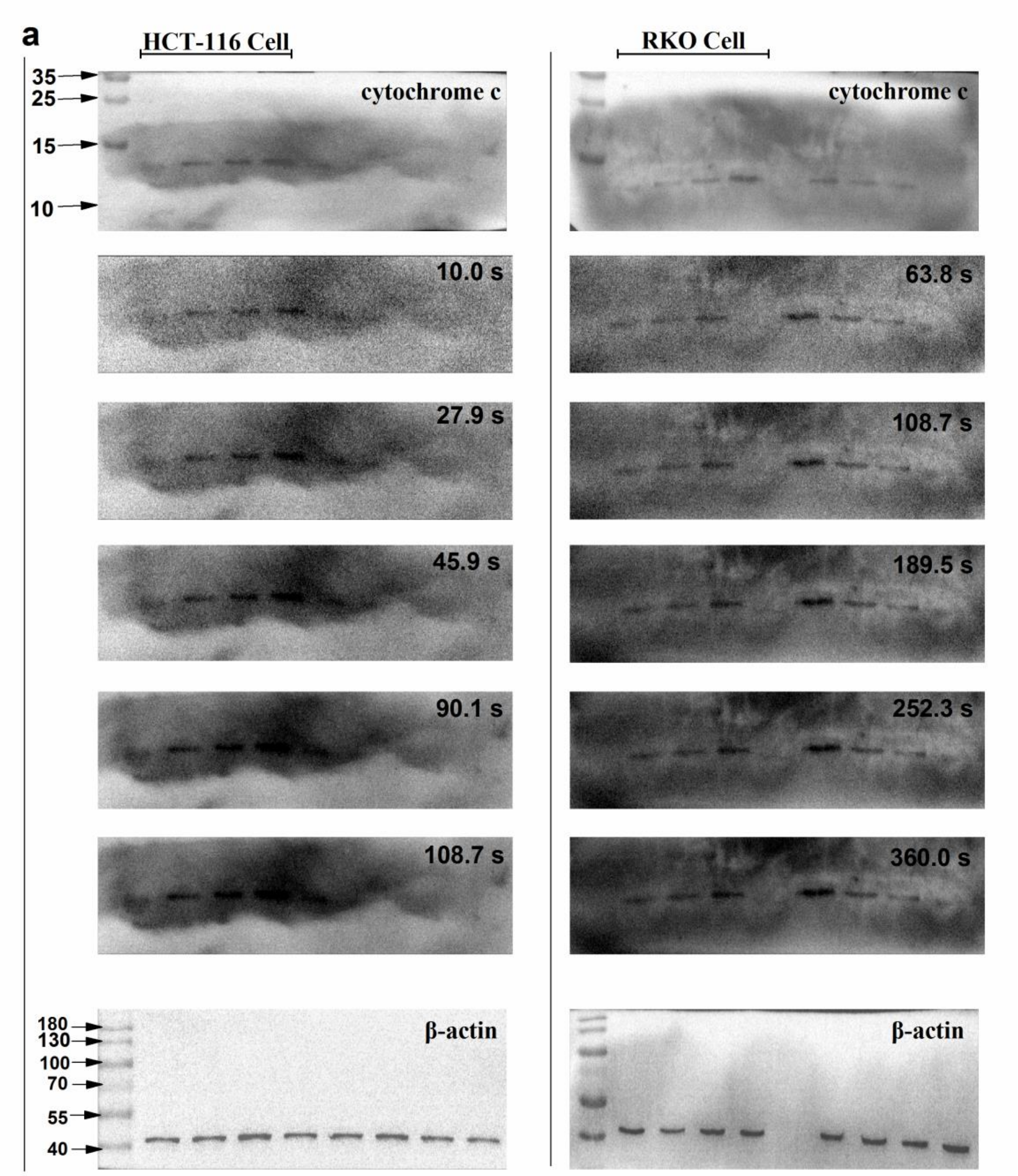

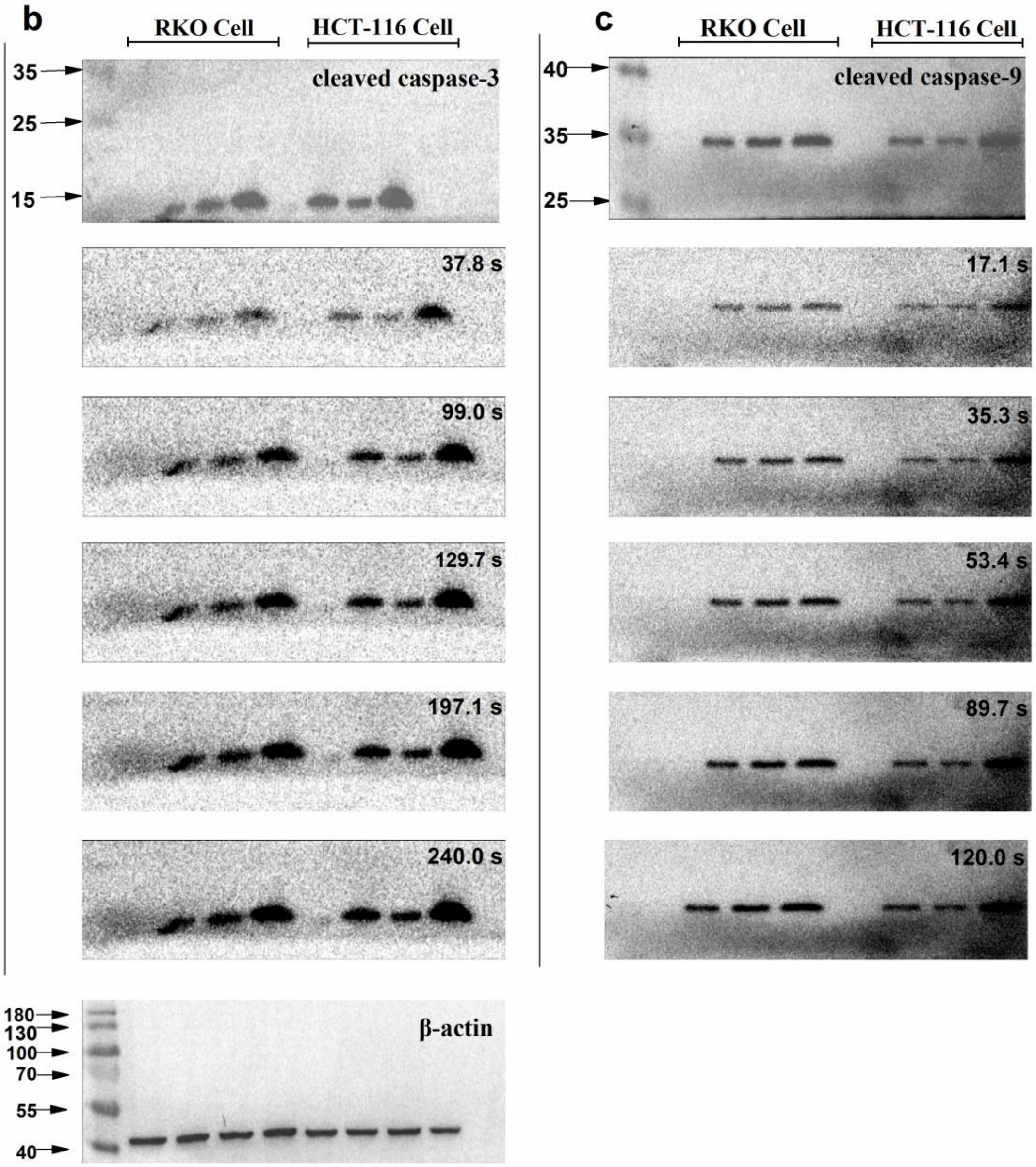
b
RKO Cell
HCT-116 Cell
35
25
15
cleaved caspase-3
37.8 s
99.0 s
129.7 s
197.1 s
240.0 s
180
130
100
70
55
40
β-actin
c
RKO Cell
HCT-116 Cell
40
35
25
cleaved caspase-9
17.1 s
35.3 s
53.4 s
89.7 s
120.0 s

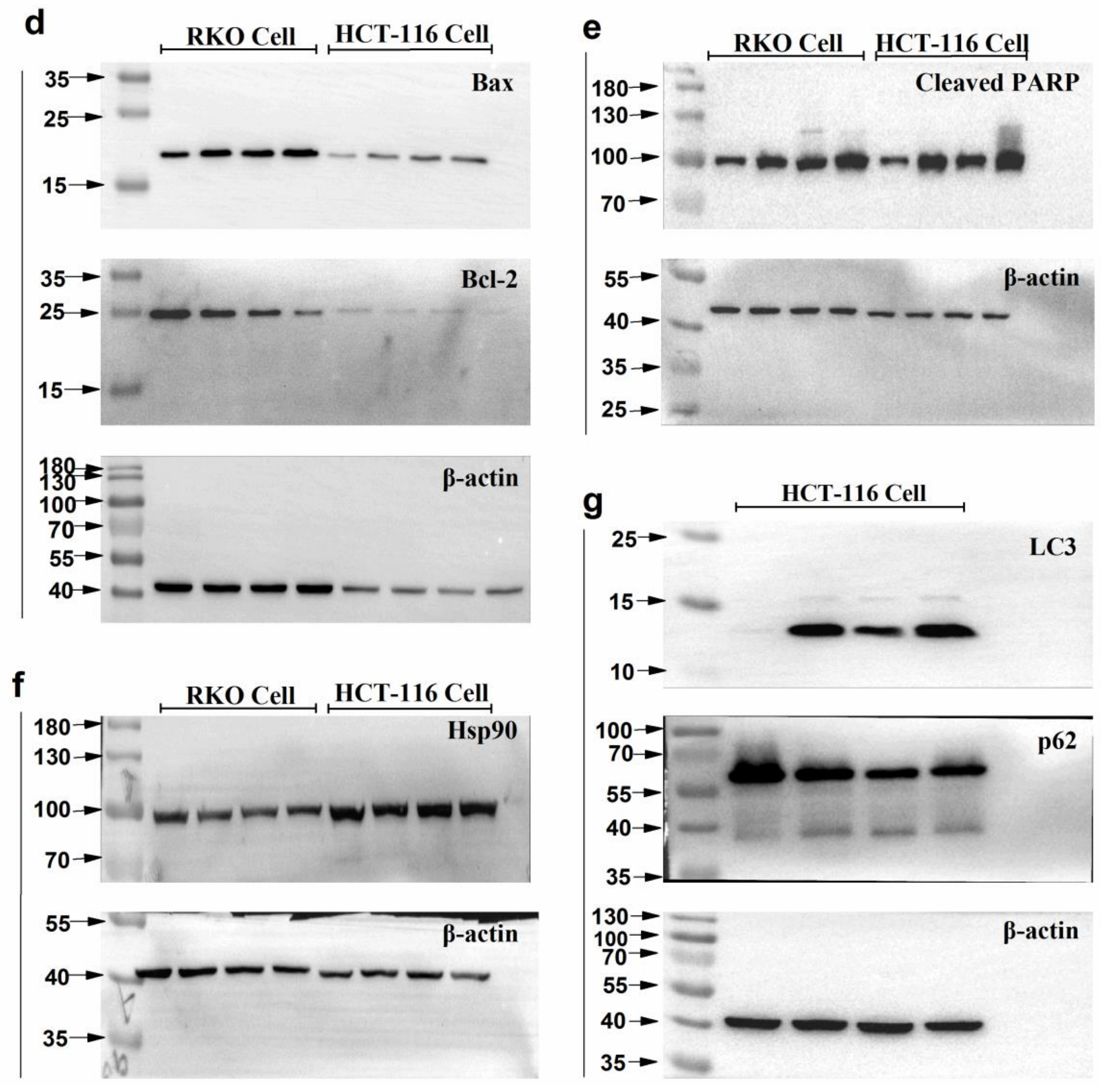

Supplementary Figure S4. **Western blots with molecular size markers for the indicated proteins in the manuscript.** (**a**), (**b**) & (**c**) Multiple exposures of cytoplasmic cytochrome c, cleaved caspase-3/9 in two CRC cell lines. (**d**), (**e**) & (**f**) Blots of Bax, Bcl-2, cleaved PARP and Hsp90 in HCT-116 and RKO cells. (**g**) Electrophoretic blots of LC3-I/II and p62 in HCT-116 cells. The blots of control, DY, 5-FU and DY+5-FU groups in each cell line are respectively presented from left to right for every indicated protein.
